# Comparison of Four Scoring Systems for Patients With Nonvariceal Upper Gastrointestinal Bleeding

**DOI:** 10.7759/cureus.74684

**Published:** 2024-11-28

**Authors:** Elrasheed M Elsabani, Badr A Badr, Mohammad Dhalaan, Anwar Alotaibi, Abdulrahman Alrujaib, Rabab Alahmed, Abdulrahman Alabbadi, Omer Kheir

**Affiliations:** 1 Hospital Medicine, Johns Hopkins Aramco Healthcare, Dhahran, SAU; 2 Internal Medicine, Johns Hopkins Aramco Healthcare, Dhahran, SAU; 3 Population Health, Johns Hopkins Aramco Healthcare, Dhahran, SAU; 4 Research, Johns Hopkins Aramco Healthcare, Dhahran, SAU; 5 Medicine, Royal College of Surgeons in Ireland – Medical University of Bahrain, Manama, BHR; 6 Health Informatics, Johns Hopkins Aramco Healthcare, Dhahran, SAU

**Keywords:** aim65, glasgow-blatchford (gbs), national early warning score + lactate (news+l), national early warning score (news), nonvariceal upper gastrointestinal bleeding

## Abstract

Introduction

Upper gastrointestinal bleeding (UGIB) is a common medical emergency that causes significant deaths and morbidity. Effective risk classification is crucial for clinical decision-making and resource allocation. Several risk assessments, including the Glasgow-Blatchford score (GBS), AIMS65, National Early Warning Score (NEWS), and National Early Warning Score + Lactate (NEWS+L), are widely used, but each has unique strengths and disadvantages. The purpose of this study is to examine the predictive performance of different scoring systems for critical outcomes, including blood transfusion requirements, inpatient admission, and 90-day mortality, in patients with nonvariceal upper GI bleeding (NVUGIB).

Method

We performed a retrospective review of 229 individuals who presented with nonvariceal upper GI hemorrhage. Baseline demographics, clinical presentations, laboratory values, and vital signs were gathered. For each patient, GBS, AIMS65, NEWS, and NEWS+L scores were calculated. The predictive accuracy of these scores for blood transfusion, inpatient admission, and 90-day mortality was evaluated using the area under the receiver operating characteristic curves (AUCs).

Results

The results show that the GBS had the highest predictive accuracy for blood transfusion (AUC: 75.7%), while NEWS was the best predictor for inpatient admission (AUC: 84.04%). For 90-day mortality, NEWS and NEWS+L performed similarly, with AUCs of 77.25% and 77.52%, respectively. AIMS65 demonstrated low predictive capacity across outcomes, although it was less successful than other ratings for specific outcomes.

Conclusion

Our results show that each risk score has distinct predictive strengths: GBS for transfusion, NEWS for admission, and NEWS/NEWS+L for mortality. Combining these scores may improve risk classification and direct-focused therapies, hence improving patient outcomes in UGIB.

## Introduction

Upper gastrointestinal bleeding (UGIB) is a medical emergency with an incidence of mortality of 5-10% [1]. It has been reported that the overall rate of mortality due to the disease ranges from 3% to 15%. These rates increase even further for those in an unstable hemodynamic condition [1-3].

Recent guidelines have recommended stratifying patients with UGIB into higher and lower risk categories for treatment decisions and prognostication [4-6]. These scoring systems have been reported to be useful in predicting mortality, rebleeding, need for transfusion, and hemostasis [7].

The International Consensus Recommendations for the Management of Patients with nonvariceal UGIB (NVUGIB) advocate for "early risk stratification" utilizing proven prognostic scores [4]. A number of scoring systems have been developed to predict the outcome of acute UGIB (AUGIB) patients, including the Rockall (RS) [8], pre-endoscopic Rockall (pre-RS) [9], Glasgow-Blatchford score (GBS) [10], AIMS65 [11,12], National Early Warning Score (NEWS) [13], and National Early Warning Score + Lactate (NEWS+L) [14]. UGIB is a global medical emergency with significant mortality and fatality rates [15]. Identifying low-risk UGIB patients who may be safely released for outpatient treatment has grown crucial in recent years, as healthcare systems worldwide encounter growing demand [16]. A variety of grading methods are used to assess the severity and prognosis of UGIB. However, these methods could vary in accuracy, simplicity of use, emergency feasibility, and therapeutic usefulness [17].

Limited data are available on the validation of scoring systems in patients with NVUGIB in Saudi Arabia. Therefore, the main aim of this study is to assess the predictive performance of multiple scoring systems for important outcomes, including blood transfusion needs, hospital admission, and 90-day mortality in patients with NVUGIB at Johns Hopkins Aramco Healthcare (JHAH) from January 2020 to September 2023.

The results of this study may have profound implications for both clinical practice and the healthcare system. From a healthcare management standpoint, the capacity to stratify patients based on projected clinical outcomes can improve the efficiency of resource allocation, decreasing unnecessary admissions and focusing intensive care resources on patients most in need.

## Materials and methods

Study design, settings, and participants

This is a retrospective, hospital-based study conducted at the JHAH facility. This facility treats employees of Arabian American Oil Company (ARAMCO) and JHAH and their dependents. The majority of the study population lived in the eastern region of Saudi Arabia in Dhahran, Al-Hasa, Ras Tanura, Abqaiq, and Udhailiyah. Eligible participants had to meet one of the following requirements: all no-trauma adults (>18 years old). Patients with UGIB admitted to the emergency department were evaluated, and the diagnosis of UGIB was based on patients’ presentations, including coffee ground vomit, hematemesis, melena, and blood in nasogastric aspirate. These patients were considered eligible for the study at the time of UGIB diagnosis. Participants were excluded if diagnosed with any type of cancer (through medical records and confirmed by biopsy results). The sample size was calculated using EPI info software (https://www.cdc.gov/epiinfo/index.html). The sample was determined to be 229 patients.

Data extraction and statistical analysis

Data were extracted from medical health records (January 2020-September 2023). A data dictionary was used, and the following variables were included: patient characteristics, vital signs, laboratory findings (hemoglobin, albumin level, blood urea nitrogen, prothrombin time (PT), and international normalized ratio (INR), comorbidities, lactate level, albumin, disposition (discharge, admission to the intensive care unit (ICU), or ward), and survival status at hospital discharge. The data were validated by selecting 10% of the data randomly and comparing it with medical health records. The data collected included patient characteristics and hemodynamic and laboratory variables at presentation necessary to calculate the GBS (Table 1), AIMS65 (Table 2), NEWS (Table 3), and NEWS+L (Table 3). All scores were calculated by a statistician using a formula in Excel (Microsoft® Corp., Redmond, WA) to calculate it.

**Table 1:** Glasgow-Blatchford score GBS: Glasgow-Blatchford score; SPB: systolic blood pressure

Glasgow-Blatchford score			
Blood urea, mmol/L		Systolic BP, mmHg	
6.5–8	2	100–109	1
8–10	3	90–99	2
10–25	4	<90	3
> 25	6	Other risk factors	
Hemoglobin, g/dL, Men		Pulse (≥100/bpm)	1
12- <13	1	Melena	1
10- <12	3	Syncope	1
<10	6	Liver disease	2
Hemoglobin, g/dL, Women		Heart failure	2
10- <12	1	Maximum score	23
<10	6		

**Table 2:** AIM65 score INR: international normalized ratio; SBP: systolic blood pressure

AIMS65 score	Value	Score
Albumin	<3.0 mg/dL	1
INR	>1.5	1
Mental status	Altered	1
SBP, mmHg	≤90	1
Age, years	≥65	1
Maximum score		5

**Table 3:** NEWS and NEWS+L scores NEWS: National Early Warning Score; NEWS+L: National Early Warning Score + Lactate; C: Centigrade; F: Fahrenheit; SPB: systolic blood pressure

NEWS	Score
Respiratory Rate	
≤8 bpm	3
9-11 bpm	1
12-20 bpm	0
> 25	2
≥25 bpm	3
Oxygen Saturations	
≤91%	3
92-93%	2
94-95%	1
≥96%	0
Any Supplemental Oxygen	
Yes	2
No	0
Temperature	
≤35°C / 95°F	3
35.1-36°C / 95.1-96.8°F	1
36.1-38°C / 96.9-100.4°F	0
38.1-39°C / 100.5-102.2°	1
≥39.1°C / 102.3°F	2
SBP, mmHg	
≤90	3
91–100	2
101-110	1
111-219	0
≥220	3
Heart Rate (bpm)	
≤40	3
41-50	1
51-90	0
91-110	1
111-130	2
≥131 bpm	3
AVPU score	
A	0
V,P orU	3
Laboratory component	
Lactate level (mmol/L)	( )
NEWS + L score	( )

Clinical characteristics and outcomes were summarized using means and standard deviations for continuous variables and frequencies and percentages for categorical variables. Receiver operating characteristic (ROC) curve analysis was used to compare the discrimination of four clinical risk scales for predicting different outcomes. The area under the curve (AUC) was calculated as a measure of discriminatory ability, with higher values indicating better accuracy. ROC curves were generated for three outcome variables: blood transfusion, inpatient admission, and 90-day mortality. Statistical analysis was conducted on RStudio (2023.06.0+421) (RStudio Team, Boston, MA) using the pROC package to generate the figures.

Ethical considerations

Ethical clearance was obtained from the JHAH Institutional Review Board (IRB# 23-47) as specified by the World Medical Association and Declaration of Helsinki.

## Results

We examined 229 patients. Slightly over half were male 122 (53.28%). On average, patients were 68.29 ± 16.05 years old, indicating a diverse age range. The most common chief complaint was abdominal pain (159, 69.43%), followed by rectal bleeding (28, 12.23%) and vomiting blood (26, 11.35%). Fewer patients presented with black or bloody stool (13, 5.68%), coughing up blood (three, 1.31%), or syncope (10, 4.37%).

Vital signs were largely stable. Systolic blood pressure averaged 129.71 ± 33.26 mmHg, and diastolic was 70.41 ± 15.31 mmHg. The heart rate was slightly high at 91.01 ± 9.25 beats per minute. The respiratory rate was 24.08 ± 16.34 breaths per minute. Oxygen saturation has an average of 98.88 ± 0.46%, with 23 (10.04%) of patients needing supplemental oxygen.

Clinical outcomes were notable. The inpatient admission rate was 179 (78.51%). Mortality was 52 (22.71%) overall and 20 (8.73%) at 90 days. Some patients had hepatic disease (27, 11.79%) and cardiac failure (49, 21.40%).

Risk scores have an average of 5.47 ± 4.26 on the GBS and 1.99 ± 0.77 on AIMS65, 1.79 ± 1.86 on NEWS, and 3.83 ± 3.26 on NEWS+L (Table 4).

**Table 4:** Demographic and clinical characteristics of the study patients (n=229) BP: blood pressure; F: Fahrenheit; GBS: Glasgow-Blatchford score; NEWS: National Early Warning Score; NEWS+L: National Early Warning Score + Lactate

Demographic/Clinical Feature	N/Mean	%/SD
Male	122	53.28%
Age (years)	68.29	16.05
Chief Complaint		
Abdominal pain	159	69.43%
Black or bloody stool	13	5.68%
Coughing up blood	3	1.31%
Rectal bleeding	28	12.23%
Vomiting blood	26	11.35%
Glasgow Coma Scale	14.1	2.39
Melaena	13	5.68%
Syncope	10	4.37%
Alert consciousness	199	86.90%
Respiratory rate (breaths/min)	24.08	16.34
Oxygen saturation (%)	98.88	0.46
Use of supplemental oxygen	23	10.04%
Systolic BP (mmHg)	129.71	33.26
Diastolic BP (mmHg)	70.41	15.31
Temperature (°F)	98.93	0.59
Heart rate (beats/min)	91.01	9.25
Blood transfusion	57	24.89%
Hepatic disease	27	11.79%
Cardiac failure	49	21.40%
Inpatient admission	179	78.51%
Mortality	52	22.71%
90-day mortality	20	8.73%
Glasgow Blatchford score	5.47	4.26
AIMS65 score	1.99	0.77
NEWS score	1.79	1.86
NEWS + L score	3.83	3.26

Several biomarkers provided insight into patients' clinical status. Hemoglobin levels averaged 11.41 ± 2.09 g/L. Albumin was within normal limits at 3.18 ± 0.58 mg/dL. Elevated blood urea of 33.64 ± 28.21 mg/dL and creatinine of 1.56 ± 1.81 mg/dL indicated possible renal impairment (Table 5).

**Table 5:** Laboratory characteristics of the study patients (n=229) INR: international normalized ratio; PT: prothrombin time

Laboratory Features	N/Mean	%/SD	Reference range
Hemoglobin (g/L)	11.41	2.09	Male: 13.8-17.2 (g/dL), Female: 12.1-15.1 (g/dL)
Albumin (mg/dL)	3.18	0.58	3.4-5.4 g/dL
Blood urea (mg/dL)	33.64	28.21	7-20 mg/dL
PT (seconds)	14.45	4.94	11.0-13.5 seconds
INR	1.24	0.43	0.8-1.1
Lactate (mmol/L)	2.04	2.04	0.5-2.2 mmol/L
Creatinine (mg/dL)	1.56	1.81	0.66-1.25 mg/dL

**Figure 1:**
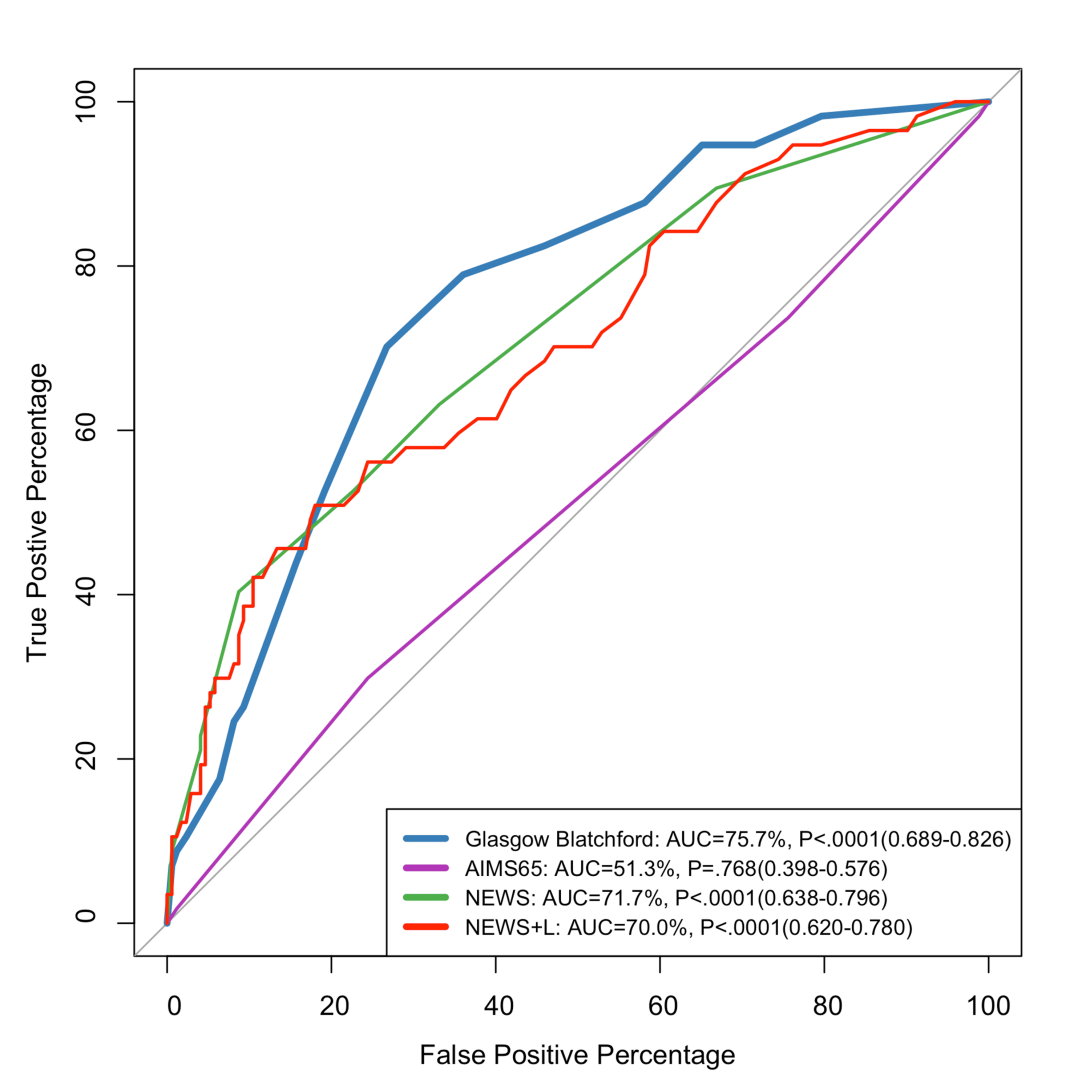
ROC curve for predicting blood transfusion ROC: receiver operating characteristic; GBS: Glasgow-Blatchford score; NEWS: National Early Warning Score; NEWS+L: National Early Warning Score + Lactate

Figure 1 compares the predictive performance of different scores for blood transfusion, inpatient admission, and 90-day mortality. The GBS had the highest discrimination for blood transfusion prediction (AUC: 75.7%).

**Figure 2:**
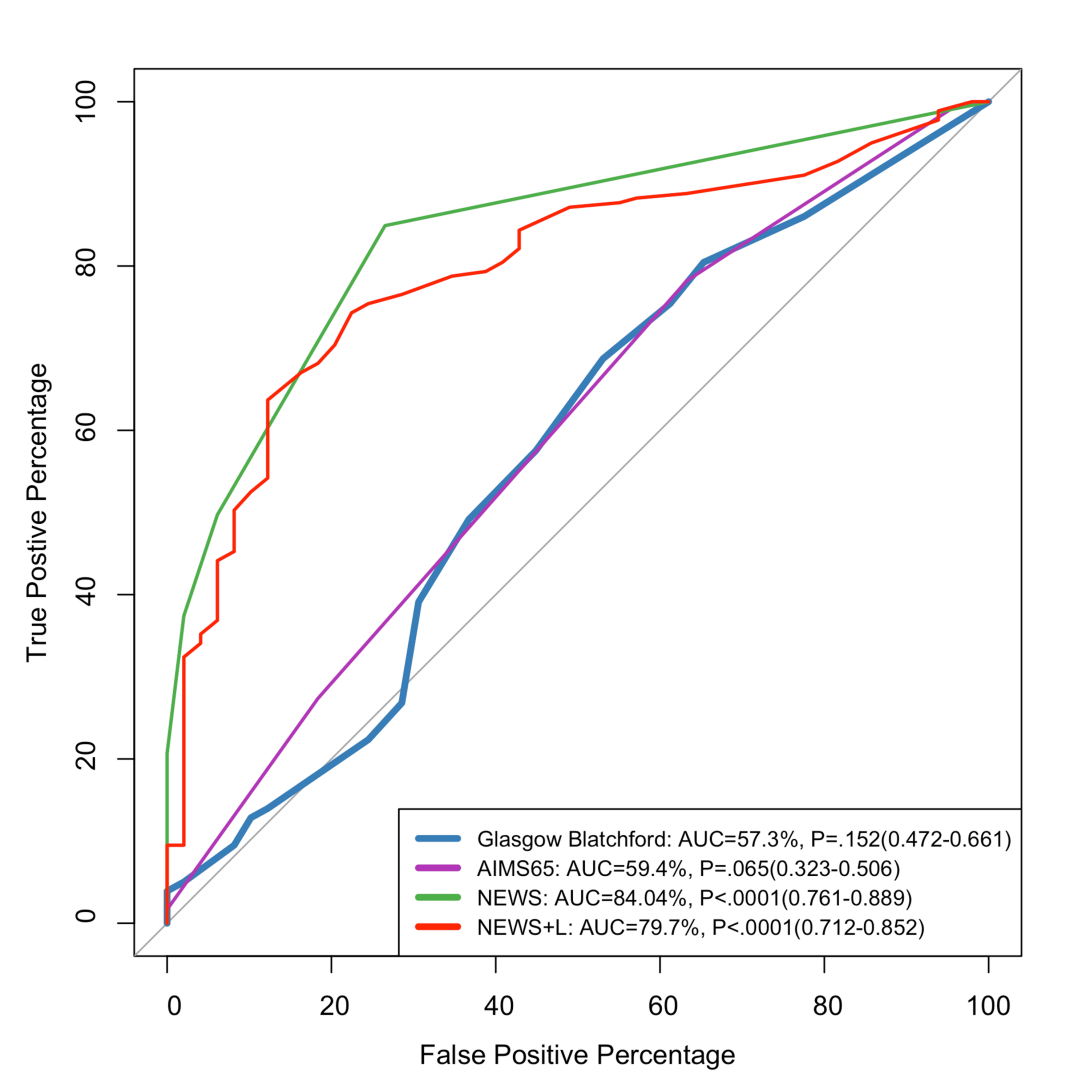
ROC curve for predicting inpatient admission ROC: receiver operating characteristic; GBS: Glasgow-Blatchford score; NEWS: National Early Warning Score; NEWS + L: National Early Warning Score + Lactate

Figure 2 shows that the NEWS scale was the best predictor for inpatient admission (84.04%).

**Figure 3:**
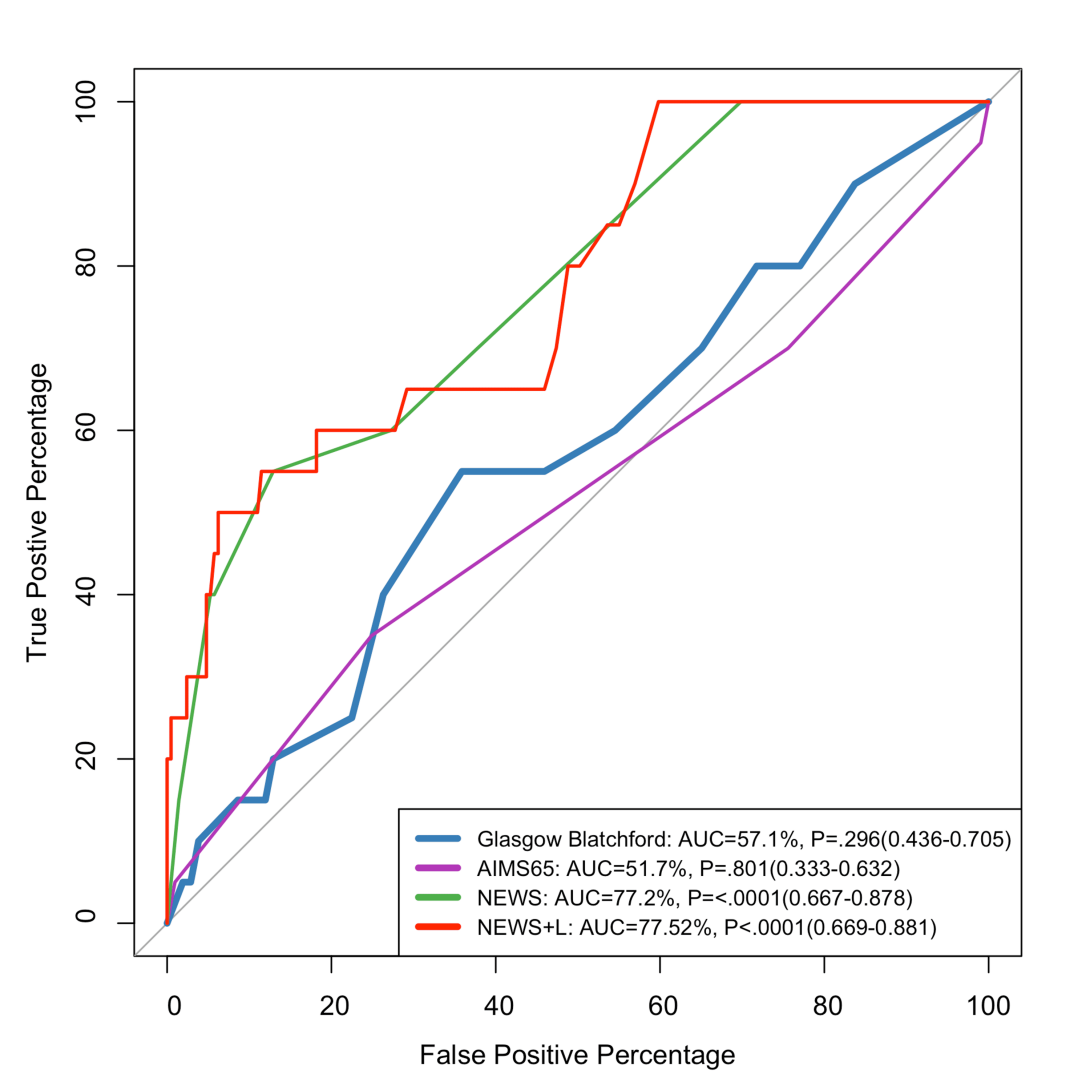
ROC curve for predicting the 90-day mortality GBS: Glasgow-Blatchford score; NEWS: National Early Warning Score; NEWS + L: National Early Warning Score + Lactate

Figure 3 indicates that NEWS and NEWS+L had the greatest ability to prognosticate 90-day mortality (77.25% and 77.52%, respectively).

Table 5 summarizes the performance of various risk assessment tools for blood transfusion, inpatient admission, and 90-day mortality. For blood transfusions, the GBS shows a sensitivity of 70.18% and specificity of 73.26%, but a low positive predictive value (PPV) of 46.51%. AIMS65 has a sensitivity of 29.82% and a specificity of 75.58%. The NEWS score has a sensitivity of 40.35% and a high specificity of 91.28%, yielding a PPV of 60.53%. In terms of inpatient admission, the NEWS tool excels with a sensitivity of 84.92% and a high PPV of 92.12%, while the GBS has a sensitivity of 68.72% but a lower specificity (46.94%). For the 90-day mortality, NEWS has a sensitivity of 55.00% and a specificity of 87.08%, with a PPV of 28.95%. Overall, the NEWS tool demonstrates the best balance of sensitivity and specificity across all outcomes, particularly for inpatient admissions.

**Table 6:** Predictive performance of risk scores for upper gastrointestinal bleeding outcomes GBS: Glasgow-Blatchford score; NEWS: National Early Warning Score; NEWS+L: National Early Warning Score + Lactate

Outcome	Risk scores	Cutoff point	Sensitivity	Specificity	PPV	NPV
Blood Transfusion	Glasgow Blatchford	7	70.18%	73.26%	46.51%	88.11%
	AIMS65	1	29.82%	75.58%	28.81%	76.47%
	NEWS	4	40.35%	91.28%	60.53%	82.20%
	NEWS+L	4.6	50.88%	81.98%	48.33%	83.43%
Inpatient Admission	Glasgow Blatchford	4	68.72%	46.94%	82.55%	29.11%
	AIMS65	2	78.21%	36.73%	81.87%	31.58%
	NEWS	1	84.92%	73.47%	92.12%	57.14%
	NEWS+L	2.5	74.30%	77.55%	92.36%	45.24%
90-Day Mortality	Glasgow Blatchford	7	55.00%	64.11%	12.79%	93.71%
	AIMS65	1	35.00%	75.12%	11.86%	92.35%
	NEWS	4	55.00%	87.08%	28.95%	95.29%
	NEWS+L	7.8	50.00%	93.78%	43.48%	95.15%

__PRESEN

## Discussion

UGIB is a common medical emergency worldwide with high morbidity and mortality rates [15]. The identification of very low-risk patients with UGIB who can be safely discharged early for outpatient management has become more important in recent years due to increasing pressure on healthcare systems around the world [16]. In this study, we assessed the predictive value of several scores for blood transfusion, hospital admission, and 90-day mortality. The 90-day mortality rate was 8.7% slightly higher than that of a previous study (3.3%) [18]. Another study showed quite similar to our results (7.7%) [19]. The comparatively high mortality rate in our study could be attributed to our sample's advanced age and major comorbidities, such as cardiac failure and hepatic illness. This high incidence emphasizes the necessity of early risk stratification, which uses several scoring systems to personalize interventions and perhaps enhance outcomes.

The GBS had the highest blood transfusion prediction compared to AIM65, NEWS, and NEWS+L. A study done by Chang et al. showed that the GBS scored 78.9% in detecting blood transfusion, which was quite similar to our results (AUC of 75.7%) [20]. The GBS takes into account characteristics such as blood urea, hemoglobin levels, and initial systolic blood pressure, all of which are important in determining the immediate risk of major bleeding and the requirement for transfusion. According to studies, the GBS is particularly useful in stratifying risk for intervention, although its ability to predict mortality may be restricted when compared to other scores [18,21].

A previous study conducted by Kim et al. [22] showed that, for in-hospital death and ICU admission rates, the AUROC values of the NEWS+L score were the highest, and these were significantly higher or comparable to those of the other risk scores (NEWS, GBS, Aim65) [22]. Our results showed that NEWS+L and NEWS had the highest values for detecting mortality compared to the GBS and AIM65. However, NEWS showed better results in detecting hospital admission than other scores. NEWS, which includes parameters such as respiration rate, oxygen saturation, and systolic blood pressure, has been recognized for its usefulness in overall clinical deterioration, making it appropriate for predicting which patients with UGIB would require hospitalization. Recent research suggests that this score, while designed for broader clinical application, is adaptive for gastrointestinal bleeding (GIB) situations, particularly in assessing overall physiological stability [23,24]. AIMS65, another frequently utilized mortality risk score, has been extensively validated for predicting outcomes in UGIB. Although it had in-between predictive value in our investigation, other studies have highlighted AIMS65's capabilities in mortality prediction due to its focus on albumin, mental status, and systemic hypotension [18,21].

Regarding, the predictive performance of different scores for blood transfusion a study conducted by Chang et al. showed that, in patients with NVUGIB, the GBS scored 78.9% in detecting the blood transfusion, which agrees with our results (AUC of 75.7%) [20]. Another study carried out among the Scottish population showed that the GBS is superior to the AIMS65 score [25].

AIM65 hospital admission prediction was lower than NEWS and NEWS+L and 59.45% and slightly higher than the GBS. A previous study showed that, in predicting the length of stay >7 days for non-variceal bleeding, AIM65 was 58.7%. These results are quite similar to our results [19].

AIM65's overall performance was not adequate in predicting the NVUGIB blood transfusion, hospital admission, and 90-day mortality; this was validated by a prior study [26].

NEWS and NEWS+L provided an overall excellent prediction for hospital admission, blood transfusion, and 90-day mortality. As for the hospital hospitalization, NEWS outperformed NEWS+L.

This study has a distinct emphasis on the Saudi population and provided useful insights into how different scoring systems operate in a group with diverse clinical and healthcare features. However, this study had several limitations. The study was a retrospective observational single study that should be addressed. The ambiguity of the ability to be generalized to other circumstances, such as different hospitals, regions, or nations, is an important concern that should be acknowledged. Data collection impairment and a lack of randomly distributed exposure should also be considered such as collecting information about endoscopy intervention types and rebreeding. Future studies could investigate the integration of these scores into a single prediction model, exploiting each's strengths to maximize clinical decision-making. Additionally, providing physicians with a superior tool for controlling upper GIB in a variety of patient categories.

## Conclusions

In conclusion, our findings demonstrate the varying efficacy of regularly used scoring systems - GBS, AIMS65, NEWS, and NEWS+L - in predicting major clinical outcomes for patients with UGIB. The GBS demonstrated improved predictive accuracy for blood transfusion requirements, affirming its importance in determining the need for prompt intervention in cases of GIB. The NEWS score had a high predictive value for inpatient admission, indicating its potential use in triaging patients for hospitalization based on their overall physiological stability. NEWS and NEWS+L beat other scores in predicting 90-day mortality, implying that these metrics, which detect symptoms of multi-organ malfunction, could be especially valuable in measuring long-term mortality risk.
